# Achieving “textbook outcome” after hepatectomy for hepatocellular carcinoma in the era of enhanced recovery: development and validation of a clinical nomogram

**DOI:** 10.3389/fonc.2026.1824197

**Published:** 2026-05-04

**Authors:** Chang Cheng, Quanyun Huang, Liping Zhu

**Affiliations:** Department of Hepatobiliary Surgery, Wuhan Hospital of Integrated Traditional Chinese and Western Medicine (Wuhan No.1 Hospital), Wuhan, Hubei, China

**Keywords:** ALBI grade, enhanced recovery after surgery, hepatectomy, hepatocellular carcinoma, nomogram, textbook outcome

## Abstract

**Background:**

Traditional perioperative metrics such as complication rates and length of stay (LOS) are fragmented and fail to capture the “ideal” recovery trajectory. “Textbook outcome” (TO) has emerged as a composite quality metric representing optimal surgical results. However, validated tools for predicting TO specifically for patients with hepatocellular carcinoma (HCC) undergoing hepatectomy within an Enhanced Recovery After Surgery (ERAS) pathway remain scarce, particularly models integrating the albumin–bilirubin (ALBI) grade.

**Methods:**

We conducted a single-center retrospective analysis of 685 consecutive patients who underwent curative hepatectomy for HCC between January 2021 and December 2025. TO was defined as the concurrent fulfillment of six criteria: no Clavien–Dindo grade ≥III complications, no prolonged LOS (>75th percentile), no readmission within 30 days, no 90-day mortality, R0 resection, and no clinically relevant bile leak. The cohort was randomly divided into a training set (*n* = 480) and a validation set (*n* = 205). Least absolute shrinkage and selection operator (LASSO) regression was utilized for variable selection. A nomogram was constructed based on multivariable logistic regression analysis. Model performance was assessed via the concordance index (*C*-index), calibration plots, and decision curve analysis (DCA).

**Results:**

The overall TO rate was 62.8% (430/685). In the training cohort, the multivariable analysis identified ALBI grade (OR = 0.45, 95%CI = 0.28–0.71), minimally invasive surgery (MIS) approach (OR = 2.15, 95%CI = 1.45–3.20), tumor size <5 cm (OR = 1.88, 95%CI = 1.22–2.90), and absence of microvascular invasion (OR = 1.65, 95%CI = 1.10–2.48) as independent predictors of achieving TO. The nomogram demonstrated good discrimination, with *C*-index values of 0.78 (95%CI = 0.73–0.83) in the training set and 0.76 (95%CI = 0.69–0.82) in the validation set. The calibration curves showed excellent agreement between the predicted and the observed probabilities. DCA confirmed the clinical utility of the nomogram across a wide range of threshold probabilities.

**Conclusion:**

TO serves as a comprehensive standard for patient-centered care. Our preliminary nomogram, which incorporates the objective ALBI grade and the surgical approach, offers a practical tool to predict ideal recovery likelihood. However, external validation is required before widespread clinical implementation.

## Introduction

The surgical management of hepatocellular carcinoma (HCC) has witnessed a paradigm shift over the past two decades. With the widespread adoption of Enhanced Recovery After Surgery (ERAS) protocols, the focus of perioperative care has evolved from merely ensuring survival to optimizing the quality of recovery and minimizing the physiological burden of surgery (1–3). Conventionally, surgical quality has been assessed using individual metrics such as postoperative morbidity, mortality, readmission rates, and length of stay (LOS). While informative, these single-point parameters are often fragmented and fail to provide a holistic view of the patient’s journey, as they may not capture the full spectrum of recovery or patient-reported outcomes (4). For instance, a patient may be discharged early (short LOS) but readmitted within a week due to complications, or may survive without severe morbidity but experience prolonged functional impairment (5). Consequently, relying on isolated outcomes may overestimate the success of surgical interventions and mask deficiencies in perioperative care systems.

To address these limitations, the concept of “textbook outcome” (TO) was introduced as a composite measure representing the “ideal” surgical result. First popularized in colorectal surgery, TO combines multiple desirable outcomes into a single “all-or-none” metric (6). A patient achieves TO only if every step of the treatment process is successful—essentially reflecting a “perfect” hospital course. Recent studies have demonstrated that TO is superior to individual complications in predicting long-term survival and quality of life in patients with gastrointestinal malignancies (7, 8). In the context of liver surgery, achieving TO implies not only technical success (R0 resection and no bile leak) but also effective perioperative management (no prolonged LOS and no readmission) (9). Despite its growing popularity in Western cohorts, data regarding TO in Asian HCC populations—who often present with distinct characteristics such as background cirrhosis and hepatitis B virus (HBV) infection—remain limited (10).

Furthermore, predicting which patients are likely to fail to achieve a TO is crucial for advancing personalized medicine in hepatic surgery (9). While there are several nomograms for predicting specific complications such as post-hepatectomy liver failure (10), a few models have been developed specifically for the composite TO endpoint in the era of ERAS (11). A significant gap in the existing models is the reliance on the Child–Pugh score to assess liver function. The Child–Pugh classification, although widely used, is limited by the subjective nature of its components (ascites and encephalopathy) and the “ceiling effect” within grade A (12). The albumin–bilirubin (ALBI) grade, an objective, evidence-based model solely based on the serum albumin and bilirubin levels, has demonstrated superior discriminatory power in assessing liver reserve and is independently associated with the achievement of TO in patients with HCC (12). However, its role in predicting short-term composite outcomes such as TO within the standardized ERAS pathways remains underexplored.

In addition, the impact of surgical approach—specifically minimally invasive surgery (MIS) including laparoscopic hepatectomy—on the achievement of TO warrants rigorous investigation. While MIS is known to reduce LOS and pain, its contribution to the strict composite definition of TO is significant, with large multicenter studies showing a higher rate of TO achievement for laparoscopic compared with open liver resection (9). However, its benefit when adjusted for complex tumor burden and liver function requires further validation in high-volume centers implementing standardized ERAS pathways.

Therefore, the primary objective of this study was to define the incidence of TO in a large cohort of patients undergoing curative hepatectomy for HCC at a tertiary center in China. Secondly, we aimed to identify independent preoperative and intraoperative predictors of TO, with a specific focus on the ALBI grade and the surgical approach. Finally, we sought to develop and validate a dynamic, user-friendly nomogram to predict the probability of achieving TO.

## Methods

### Study design and population

This is a single-center, retrospective cohort study conducted at the Department of Hepatobiliary Surgery of our hospital. This study is reported in accordance with the TRIPOD (Transparent Reporting of a Multivariable Prediction Model for Individual Prognosis Or Diagnosis) statement (13). We reviewed the medical records of all consecutive patients who underwent hepatectomy for histopathologically confirmed HCC between January 1, 2021, and December 31, 2025. The inclusion criteria were: 1) age ≥18 years; 2) elective curative liver resection (defined as complete macroscopic removal of the tumor); 3) Child–Pugh class A or B liver function; and 4) management under the institutional standard ERAS protocol (3). The exclusion criteria were: 1) emergency surgery for ruptured HCC; 2) palliative resection or R2 resection; 3) concomitant ablation or other organ resection (e.g., colectomy); 4) clinical evidence of distant metastasis; and 5) missing data >10% for key variables.

The study protocol was approved by the Institutional Review Board and complied with the Declaration of Helsinki. Informed consent was waived due to the retrospective nature of the study.

### ERAS protocol and surgical technique

All patients were managed according to a standardized ERAS pathway adapted from the ERAS Society guidelines for liver surgery (3). Key components included preoperative counseling, carbohydrate loading, avoidance of prolonged fasting, goal-directed fluid therapy, multimodal opioid-sparing analgesia, early removal of drains and catheters, and early mobilization/feeding. Adherence to the ERAS protocol was prospectively audited, with an overall institutional compliance rate of >80% required for patient inclusion in the standard pathway. Hepatectomies were performed by three senior consultant surgeons using consistent techniques. The approach (open *vs*. minimally invasive) was determined based on tumor location, size, and surgeon preference, although MIS was prioritized whenever feasible, specifically for tumors <5 cm located in anterolateral segments without major vascular involvement.

### Definition of textbook outcome

Based on international consensus and previous literature on composite quality measures in liver surgery (14, 15), TO was defined as the composite fulfillment of the following six parameters: 1) no postoperative severe complications: defined as Clavien–Dindo grade <III (16); 2) no prolonged LOS: LOS ≤75th percentile of the cohort (determined to be ≤10 days in this study); 3) no readmission: no unplanned readmission within 30 days of discharge; 4) no mortality: no death within 90 days after surgery; 5) R0 resection: microscopically negative surgical margins; and 6) no clinically relevant bile leak: absence of grade B or C bile leak according to the International Study Group of Liver Surgery (ISGLS) definition (17).

### Data collection and variable definitions

Data were extracted from the electronic medical record system. Preoperative variables included age, gender, body mass index (BMI), comorbidities (Charlson comorbidity index, CCI), hepatitis B/C status, and laboratory values. The ALBI score was calculated using the formula: (log10 bilirubin [µmol/L] × 0.66) + (albumin [g/L] × −0.085) (18). Patients were stratified into ALBI grade 1 (less than or equal to −2.60), grade 2 (greater than −2.60 to less than or equal to −1.39), and grade 3 (greater than −1.39). Intraoperative variables included surgical approach (open *vs*. laparoscopic/robotic), extent of resection (major: three or more segments *vs*. minor: less than three segments), operative time, and estimated blood loss (EBL). Pathological data included tumor size (maximum diameter), number of tumors, and microvascular invasion (MVI).

### Statistical analysis

Statistical analyses were performed using R software version 4.5.1 (The R Foundation for Statistical Computing). Multiple imputation by chained equations (MICE) was used for missing data (<5% for all variables). Missing data were assumed to be missing at random and handled using predictive mean matching within the MICE framework. The cohort was randomly split into a training set (70%) and a validation set (30%) (19).

Continuous variables were compared using the Mann–Whitney *U* test or *t*-test, while categorical variables were compared using the chi-square test or Fisher’s exact test. To avoid overfitting and to handle multicollinearity, least absolute shrinkage and selection operator (LASSO) regression was applied in the training set to screen non-zero coefficient variables. The optimal penalization coefficient lambda (*λ*) for LASSO was selected using 10-fold cross-validation based on the minimum binomial deviance. The variables selected by LASSO were entered into a multivariable logistic regression model to identify independent predictors of TO.

A nomogram was constructed based on the multivariable model. The model’s discrimination was quantified by the concordance index (*C*-index) and the area under the receiver operating characteristic (ROC) curve (AUC). Calibration was assessed using calibration plots with 1,000 bootstrap resamples and the Hosmer–Lemeshow test. Clinical utility was evaluated using decision curve analysis (DCA) to calculate the net benefit at different threshold probabilities. A two-sided *p*-value <0.05 was considered statistically significant.

## Results

### Patient characteristics and TO incidence

**Figure 1:**
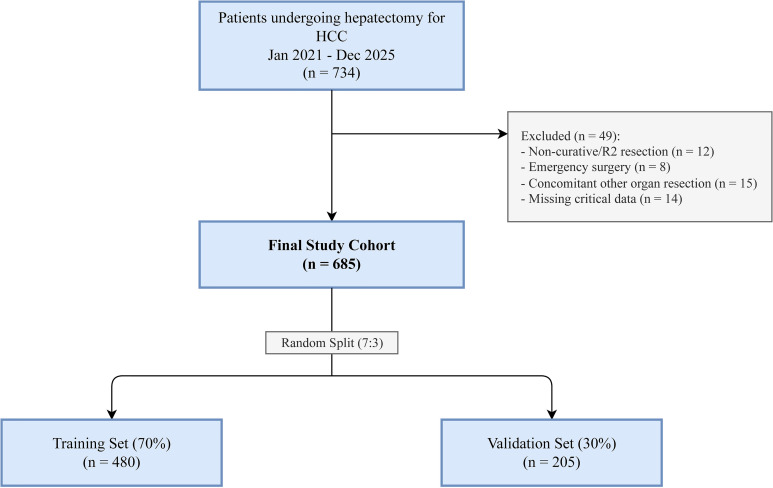
Study flowchart. diagram illustrating the inclusion and exclusion process of the study cohort, leading to the final sample size of 685 patients.

A total of 734 patients were screened, with 685 patients meeting the inclusion criteria. The flowchart of patient selection is shown in **Figure 1**. The median age was 58 years [interquartile range (IQR) = 49–66 years], and 520 (75.9%) were men. The majority of patients (82.3%) had HBV infection. The median ALBI score was −2.71, with 405 (59.1%) patients classified as ALBI grade 1 and 280 (40.9%) as ALBI grade 2.

**Figure 2:**
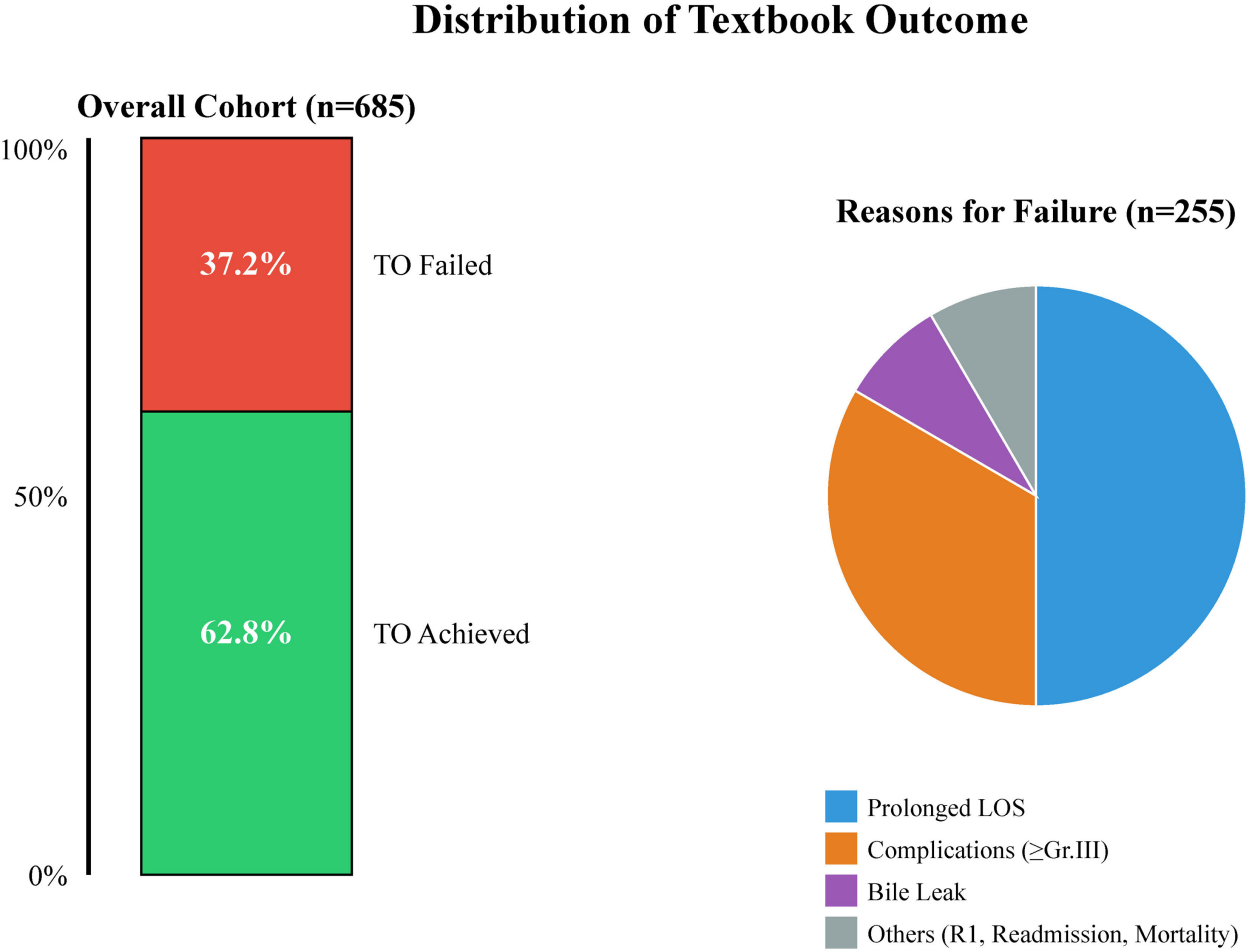
Distribution of the textbook outcome (TO) parameters. A stacked bar chart showing the frequency of achieving the individual TO components. The *inset* pie chart displays the primary reasons for failure to achieve TO among the non-TO group.

The overall rate of TO was 62.8% (430/685). Among the 255 patients who failed to achieve TO, the specific reasons for failure (non-mutually exclusive) were: prolonged LOS (18.5%), grade ≥III complications (12.4%), clinically relevant bile leak (5.8%), non-R0 resection (4.1%), readmission (3.2%), and mortality (0.6%). The distribution of the TO components is visualized in **Figure 2**.

The baseline characteristics stratified by TO status are presented in **Table 1**. Patients in the TO group had significantly lower ALBI scores (higher proportion of grade 1: 68.1% *vs*. 43.9%, *p* < 0.001), smaller tumor size (median: 4.2 *vs*. 6.5 cm, *p* < 0.001), and were more likely to undergo MIS (60.2% *vs*. 35.7%, *p* < 0.001) compared with those in the non-TO group.

**Table 1:** Baseline characteristics of patients stratified by textbook outcome (TO) status (N=685).

Variable	Total (N=685)	TO group (n=430)	Non-TO group (n=255)	P Value
Age (years), median (IQR)	58 (49-66)	57 (48-65)	60 (51-68)	0.042
Gender, Male, n (%)	520 (75.9)	328 (76.3)	192 (75.3)	0.765
BMI (kg/m²), median (IQR)	23.5 (21.4-25.8)	23.4 (21.2-25.5)	23.8 (21.8-26.2)	0.210
ASA Grade ≥ III, n (%)	145 (21.2)	82 (19.1)	63 (24.7)	0.081
Hepatitis B Surface Antigen (+), n (%)	564 (82.3)	355 (82.6)	209 (82.0)	0.844
ALBI Grade, n (%)				<0.001
Grade 1	405 (59.1)	293 (68.1)	112 (43.9)	
Grade 2	280 (40.9)	137 (31.9)	143 (56.1)	
Child-Pugh Class A, n (%)	648 (94.6)	412 (95.8)	236 (92.5)	0.068
Surgical Approach, n (%)				<0.001
Open	335 (48.9)	171 (39.8)	164 (64.3)	
Minimally Invasive (Lap/Robotic)	350 (51.1)	259 (60.2)	91 (35.7)	
Major Hepatectomy (≥3 segments), n (%)	210 (30.7)	105 (24.4)	105 (41.2)	<0.001
Operative Time (min), median (IQR)	185 (140-230)	170 (135-215)	210 (160-260)	<0.001
Estimated Blood Loss (mL), median (IQR)	200 (100-400)	150 (100-300)	300 (150-600)	<0.001
Tumor Size (cm), median (IQR)	5.0 (3.2-7.5)	4.2 (2.8-6.1)	6.5 (4.5-9.2)	<0.001
Microvascular Invasion (MVI), n (%)	225 (32.8)	118 (27.4)	107 (42.0)	<0.001

TO, Textbook Outcome; BMI, Body Mass Index; ASA, American Society of Anesthesiologists; ALBI, Albumin-Bilirubin; IQR, Interquartile Range.

### Predictor selection and model development

**Figure 3:**
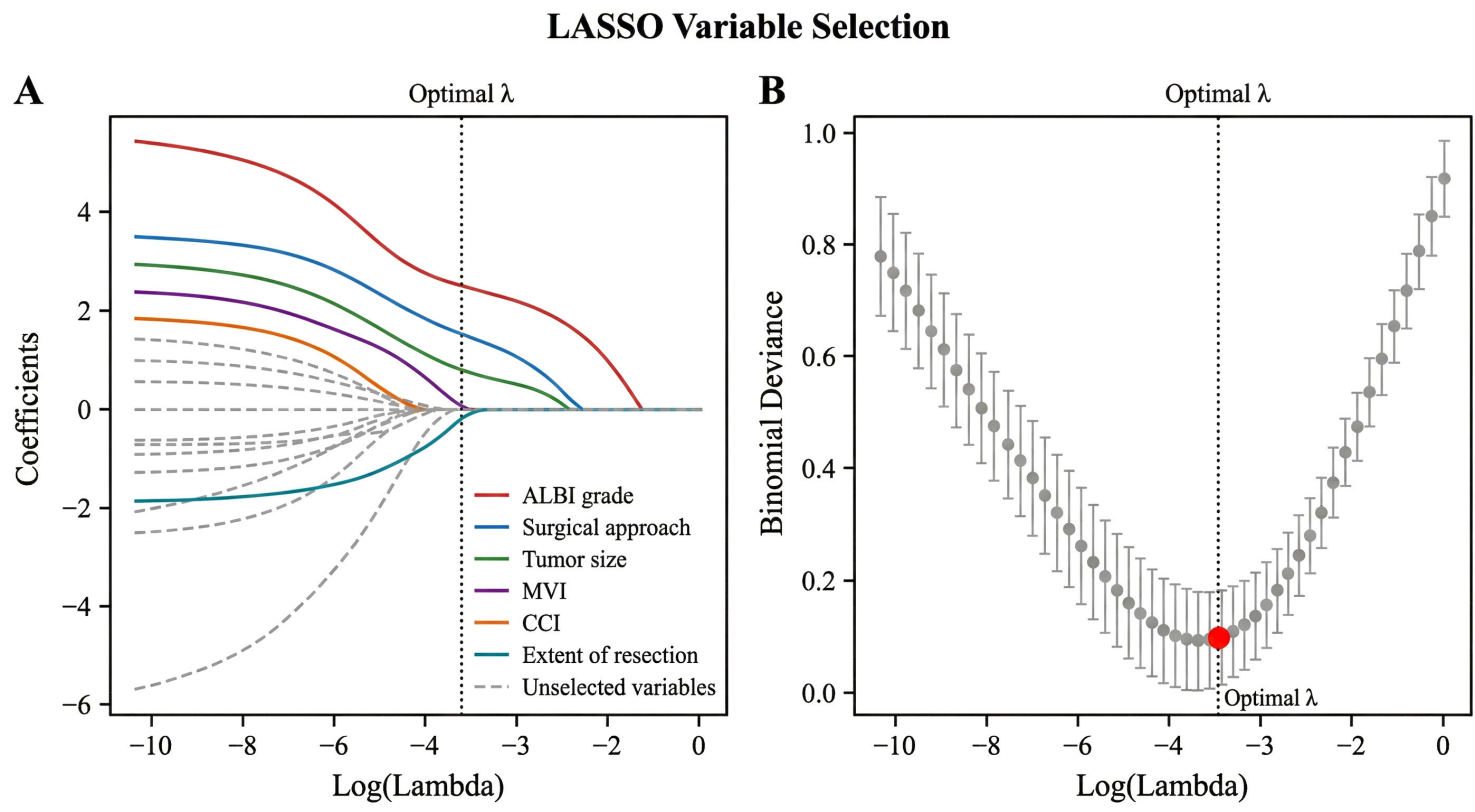
Least absolute shrinkage and selection operator (LASSO) regression analysis. **(A)** LASSO coefficient profiles of the 18 candidate variables. **(B)** Selection of the optimal penalization coefficient lambda (*λ*) using 10-fold cross-validation. *Dotted vertical lines* represent the optimal *λ* value.

In the training cohort (*n* = 480), 18 potential variables were initially included. LASSO regression analysis identified six features with non-zero coefficients at the optimal lambda value (*λ* = 0.023): ALBI grade, CCI, surgical approach, tumor size, MVI, and extent of resection (**Figure 3**).

These variables were entered into the multivariable logistic regression analysis. The results showed that ALBI grade 2 (*vs*. grade 1: OR = 0.45, 95%CI = 0.28–0.71, *p* = 0.001), open surgery (*vs*. MIS: OR = 0.47, 95%CI = 0.31–0.69, *p* < 0.001), tumor size ≥5 cm (OR = 0.53, 95%CI = 0.34–0.82, *p* = 0.004), and presence of MVI (OR = 0.61, 95%CI = 0.40–0.91, *p* = 0.016) were independent negative predictors of achieving TO (**Table 2**). Conversely, MIS and better liver function (ALBI grade 1) were strongly associated with achieving TO.

**Table 2:** Univariable and multivariable logistic regression analysis of factors associated with achieving textbook outcome in the training cohort (n=480).

Variable	Univariable analysis		Multivariable analysis	
	OR (95% CI)	P Value	OR (95% CI)	P Value
Age (>60 vs ≤60)	0.92 (0.68-1.25)	0.582	–	–
Gender (Male vs Female)	1.05 (0.70-1.58)	0.814	–	–
CCI Score (>1 vs 0-1)	0.72 (0.48-1.08)	0.112	0.88 (0.55-1.42)	0.612
ALBI Grade (2 vs 1)	0.38 (0.26-0.56)	<0.001	0.45 (0.28-0.71)	0.001
Surgical Approach (Open vs MIS)	0.35 (0.24-0.51)	<0.001	0.47 (0.31-0.69)	<0.001
Extent of Resection (Major vs Minor)	0.48 (0.32-0.72)	<0.001	0.75 (0.45-1.24)	0.263
Tumor Size (≥5cm vs <5cm)	0.42 (0.29-0.61)	<0.001	0.53 (0.34-0.82)	0.004
MVI (Present vs Absent)	0.51 (0.35-0.75)	<0.001	0.61 (0.40-0.91)	0.016

Variables selected by LASSO regression were included in the multivariable model. OR > 1 indicates a higher likelihood of achieving TO, but here negative factors are shown as risk factors (<1) for clarity, or can be interpreted inversely. In this table, the reference groups are the "Good" outcomes (e.g., ALBI 1, MIS). ORs < 1 indicate a reduced chance of achieving TO.

### Nomogram construction and validation

**Figure 4:**
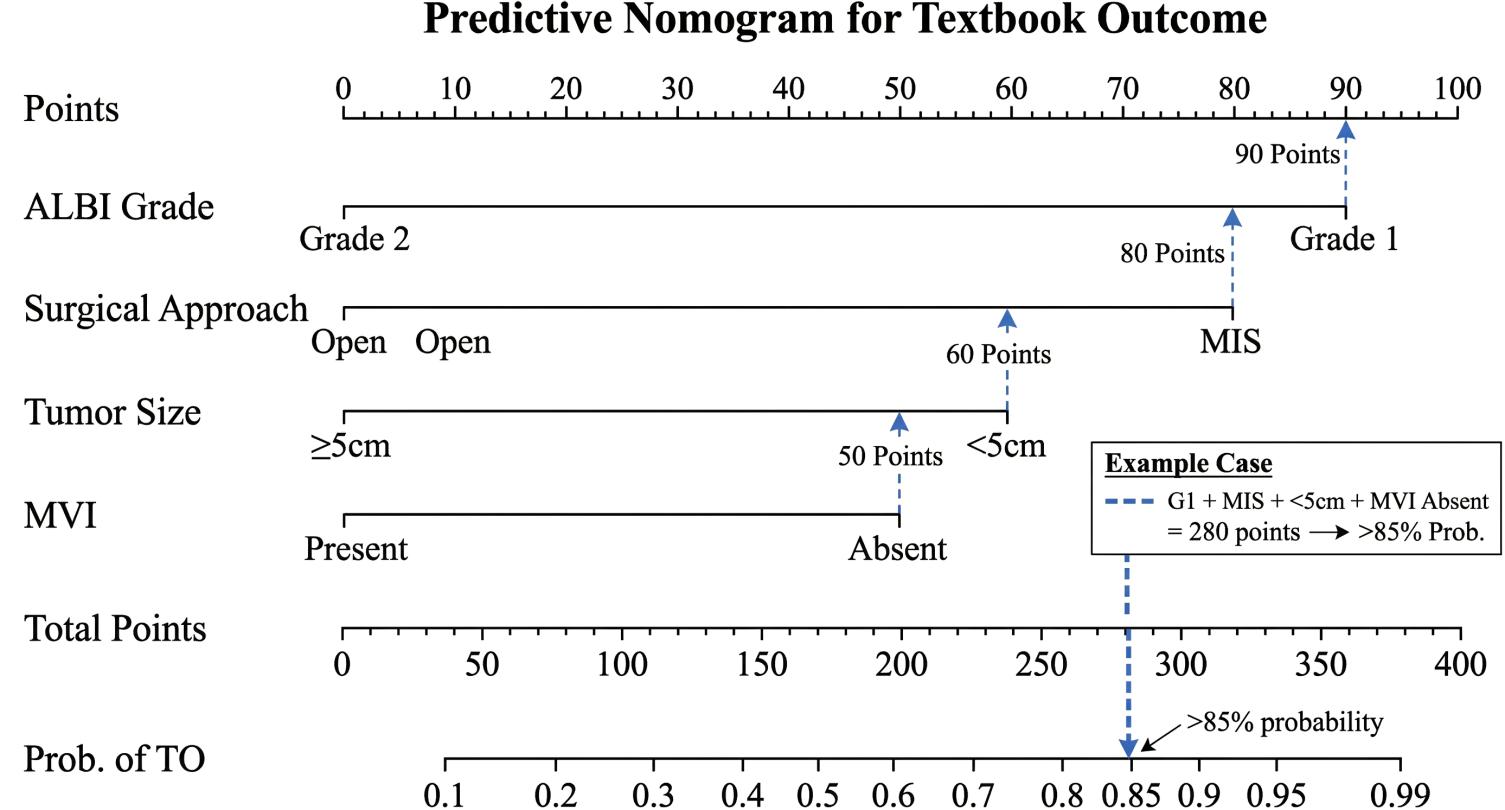
Predictive nomogram for textbook outcome (TO). A nomogram incorporating the albumin–bilirubin (ALBI) grade, surgical approach, tumor size, and microvascular invasion (MVI) to predict the probability of achieving TO. To use: locate each variable on its axis, draw a vertical line to the “Points” scale, sum the points, and draw a line from “Total Points” to the “Probability of TO” scale.

Based on the multivariable analysis, a nomogram was developed to predict the individual probability of achieving TO (**Figure 4**). The nomogram assigns points to each of the four independent factors (i.e., ALBI grade, surgical approach, tumor size, and MVI). For example, a patient with ALBI grade 1 undergoing laparoscopic hepatectomy for a 3-cm tumor without MVI would receive a high total score, corresponding to a >85% probability of achieving TO.

#### Discrimination

**Figure 5:**
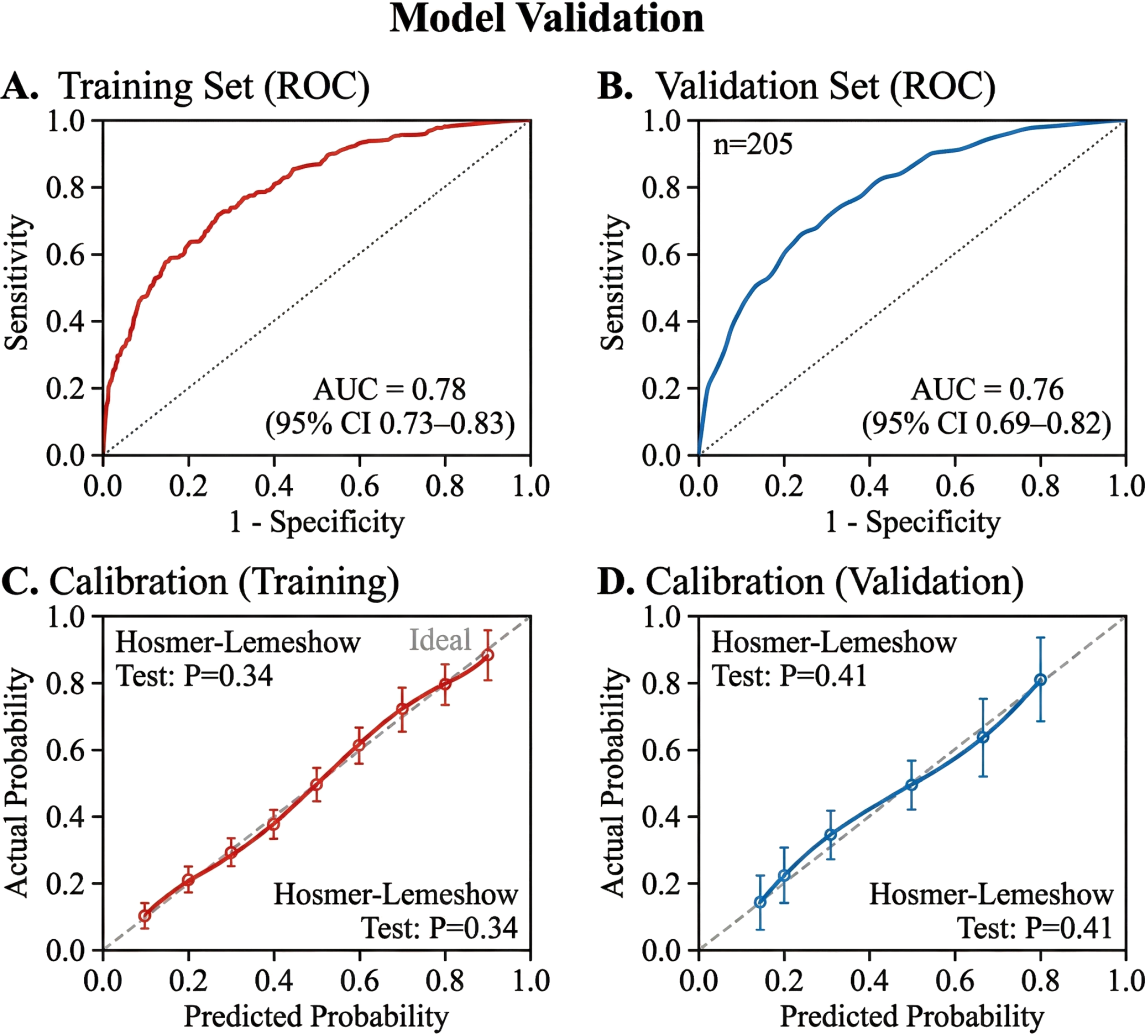
Model validation. **(A, B)** Receiver operating characteristic (ROC) curves for the training cohort [area under the curve (AUC) = 0.78] **(A)** and the validation cohort (AUC = 0.76) **(B)**. **(C, D)** Calibration plots for the training cohort **(C)** and the validation cohort **(D)**. The *diagonal line* represents perfect prediction.

In the training set, the *C*-index of the nomogram was 0.78 (95%CI = 0.73–0.83). In the validation set (*n* = 205), the *C*-index remained robust at 0.76 (95%CI = 0.69–0.82), indicating good predictive accuracy. The ROC curves for both sets are shown in **Figures 5A, B**.

#### Calibration

The calibration plots for both the training and validation cohorts demonstrated optimal agreement between the nomogram-predicted probabilities and the actual observed TO rates, with the bias-corrected lines close to the ideal 45-degree diagonal line (**Figures 5C, D**). The Hosmer–Lemeshow test yielded non-significant *p*-values (*p* = 0.34 and *p* = 0.41, respectively), suggesting good goodness-of-fit.

#### Clinical utility

**Figure 6:**
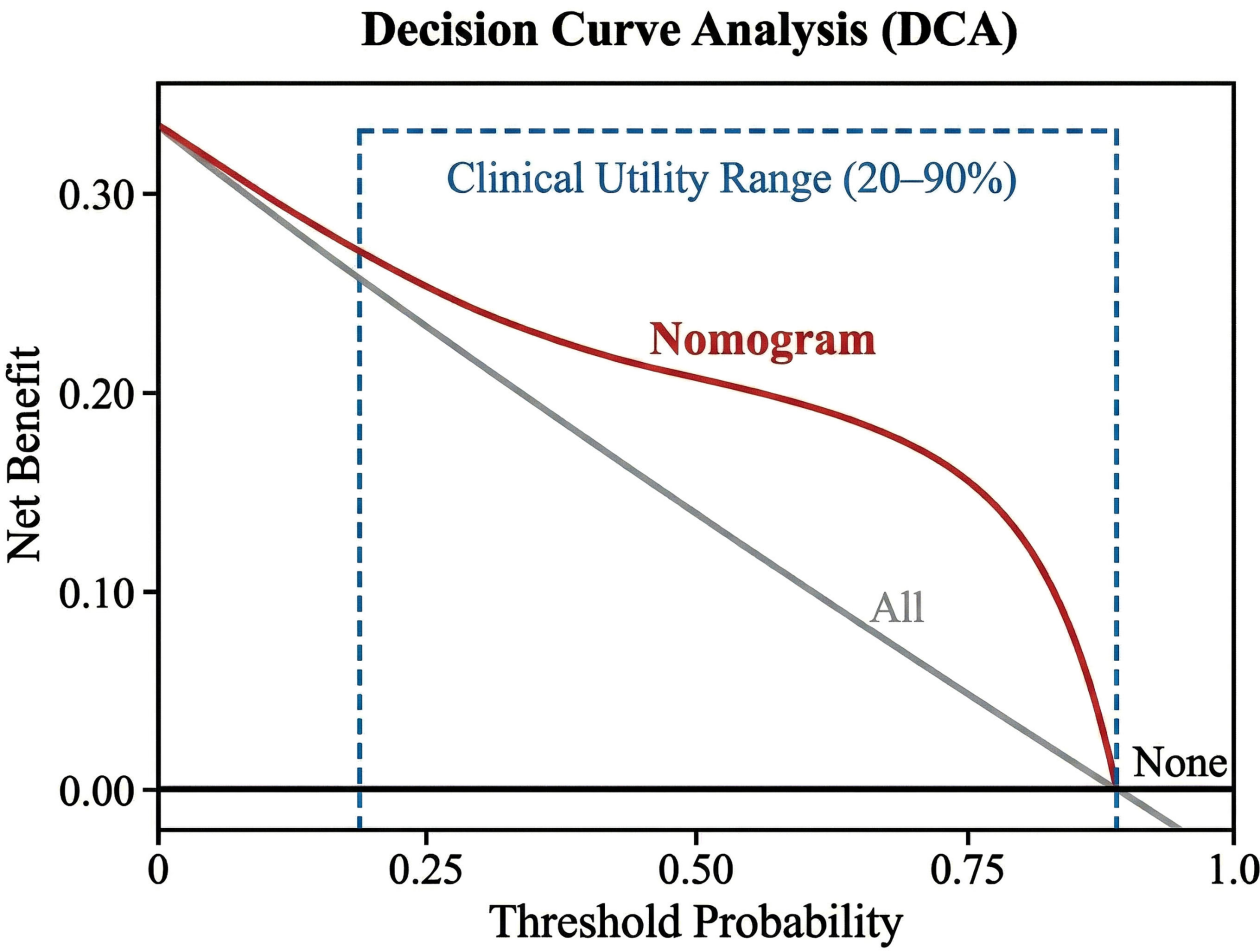
Decision curve analysis (DCA). The *y*-axis measures the net benefit. The *red line* represents the nomogram. The *gray line* represents the assumption that all patients achieve textbook outcome (TO), while the *black line* represents the assumption that no patients achieve TO. The decision curve shows that using the nomogram adds net benefit compared with treat-all or treat-none strategies.

The DCA showed that using the nomogram to predict TO provided a greater net benefit than “treating all” or “treating none” schemes across a threshold probability range of 20%–90% (**Figure 6**).

## Discussion

In the present study, we utilized a composite endpoint, “textbook outcome,” to comprehensively evaluate the quality of surgical care for patients with HCC in the context of an established ERAS program (12). We observed a TO rate of 62.8%, which compares favorably with recent international benchmarks ranging from 40% to 60% (20, 21). More importantly, we successfully developed and validated a novel nomogram that incorporates the ALBI grade and the surgical approach. This tool demonstrated robust discrimination and calibration, providing clinicians with a practical instrument to identify patients at high risk of failing to achieve an ideal recovery.

The paradigm shift from “saving lives” to “optimizing recovery” necessitates new metrics that capture the multidimensional nature of surgical success, moving beyond single endpoints (22). TO serves as such a composite measure, with recent studies highlighting its value in quality assessment and as a predictor of survival (9). For instance, a large international multicenter study in liver surgery demonstrated that TO is a valuable tool for assessing patient-level hospital performance and is associated with favorable postoperative outcomes (9). The TO rate of 62.8% observed in our center aligns with the rates reported in contemporary benchmark studies, a finding that may be attributed to high procedural volume and the systematic implementation of enhanced recovery protocols (9). It is important to acknowledge that our TO definition utilized an LOS threshold of the 75th percentile (≤10 days), which is cohort-dependent. While some previous liver surgery studies used fixed cutoffs (e.g., ≤5 or 7 days), a percentile-based approach better reflects the specific discharge practices and ERAS context of our institution. However, this may limit direct comparability with cohorts from other centers with different baseline LOS distributions. However, prolonged LOS remained the most frequent reason for failure in our cohort (18.5%). This underscores that, even in high-performing centers, non-medical and system-related factors—such as discharge logistics, patient preparedness, and socioeconomic disparities—can significantly impact the achievement of an ideal recovery pathway (22). Furthermore, it is critical to acknowledge the inherent limitations of the TO metric itself. As an “all-or-none” composite measure, TO does not distinguish between a patient who narrowly misses a single parameter (e.g., a marginal prolongation of LOS due to logistical reasons) and a patient who experiences multiple severe clinical complications. This binary nature may inadvertently mask partial but clinically meaningful recovery trajectories, highlighting the need to interpret TO in conjunction with individual clinical parameters.

A key innovation of our model is the integration of the ALBI grade. While the Child–Pugh score is the traditional standard, it is limited by subjective variables such as the assessment of ascites and encephalopathy (18). Our multivariable analysis confirmed that ALBI grade 2 was a strong independent predictor of treatment outcome failure (OR = 0.45), a finding consistent with studies identifying the ALBI grade as an independent predictor of post-hepatectomy liver failure and other complications (23). This supports the growing body of evidence that ALBI provides a more objective and granular assessment of liver reserve compared with the Child–Pugh classification (24), as also highlighted in the work by Hiraoka et al. on refining hepatic function assessment (25). Even within the so-called safe Child–Pugh A group, patients with higher ALBI scores (grade 2) have subclinical hepatic dysfunction that impairs protein synthesis and metabolic clearance, leading to increased risks of ascites, infection, and prolonged LOS—all of which negate successful treatment outcomes, as evidenced by its role as an independent risk factor for mortality (26).

Furthermore, our study reinforces the pivotal role of MIS in achieving TO. Patients undergoing laparoscopic or robotic hepatectomy have demonstrated a significantly higher likelihood of achieving TO compared with open surgery, with large multicenter analyses reporting superior achievement rates (9). This benefit persists as an independent factor even after adjusting for confounding variables such as tumor size and location. The physiological advantages of MIS, including a reduced postoperative inflammatory response and a lower incidence of complications such as acute kidney injury, contribute to less pain and facilitate earlier mobilization, which are cornerstones of ERAS protocols (27). Recent studies from 2024 have also corroborated that MIS is a significant facilitator of TO in liver surgery (28). However, it is crucial to note that technical complexity remains a significant barrier. Large tumor size (e.g., >5 cm) is associated with more aggressive disease and increases the risk of R1 resection, directly impacting the “oncological” component of TO (29). Similarly, factors such as MVI and complex tumor location can elevate the risk of surgical complications such as bile leaks, affecting the “surgical” quality components.

The clinical utility of our nomogram lies in preoperative decision-making. By calculating the probability of TO, surgeons can stratify patients. For a patient with a low predicted probability (e.g., <40%), “pre-habilitation” strategies should be aggressively implemented. This could include nutritional support to improve the albumin levels (and thus the ALBI score) (30), intensive cardiopulmonary exercise (31), and psychological counseling to address anxiety (32). In addition, for borderline resectable cases with low TO probability, the nomogram might prompt a reconsideration of the surgical approach or the use of neoadjuvant therapies (33, 34).

There are several limitations to this study. Firstly, its retrospective nature introduces inherent selection bias, although we attempted to mitigate this using multivariable analysis and strict inclusion criteria. Specifically, the choice of surgical approach (MIS *vs*. open) was influenced by tumor characteristics and surgeon preference, meaning that patients selected for MIS likely had more favorable baseline profiles. Furthermore, while patients were managed according to an ERAS pathway, individual compliance rates and the potential confounding effect of differing surgeon experience levels were not fully integrated into our model, which may influence TO achievement independent of the included variables. Secondly, as a single-center study in China with a predominance of HBV-related HCC, the generalizability of our nomogram to Western populations with hepatitis C virus (HCV) or non-alcoholic steatohepatitis/metabolic dysfunction-associated steatohepatitis (NASH/MASLD) etiologies requires external validation (35). Crucially, our nomogram lacks external validation. Because the model was developed and internally validated on a specific patient population, its performance and generalizability remain uncertain in broader settings. External validation using independent datasets is essential before this prediction model can be routinely implemented in clinical practice. Thirdly, we did not include genomic data, which might further refine the prediction of biological recurrence.

## Conclusion

In conclusion, achieving TO represents a comprehensive standard for patient-centered care. Our preliminary nomogram, which incorporates the objective ALBI grade and the surgical approach, offers a practical tool to predict ideal recovery likelihood, facilitate personalized perioperative management, and help identify patients who would benefit most from targeted pre-habilitation interventions. However, given the single-center retrospective nature of this study, external validation is required before this model can be recommended for widespread clinical implementation.

## Data Availability

The original contributions presented in the study are included in the article/Supplementary Material. Further inquiries can be directed to the corresponding author.
